# The early Impact of stress related to COVID-19 Pandemic on physicians in Tunisia

**DOI:** 10.1192/j.eurpsy.2022.863

**Published:** 2022-09-01

**Authors:** N. Sayari, S. Ellini, C. Wissal, S. Halayem, M. Cheour, R. Damak

**Affiliations:** Razi Hospital, Ibn Omran Departement, manouba, Tunisia

**Keywords:** Preventative Medicine, pandemic, Stress, Covid-19

## Abstract

**Introduction:**

Contagious disease outbreaks can have major repercussions on medical stuff. Doctors in Tunisia were requested to act as the first-line filter in managing active cases during the beginning of COVID19 pandemic.

**Objectives:**

This study aims to assess perceived stress in Tunisian doctors during COVID19 pandemic early stages and to identify factors associated to stress in order to guide prevention strategies.

**Methods:**

This was a cross-sectional study conducted through an online survey, from April 18th 2020 to June 6th 2020. A 62-item semi-structured survey was created, consisting of 5 series of questions and scales. Linear regression models were performed to identify the associations between the study variables and the perceived stress score of the participants.

**Results:**

We included 203 physicians in this study. Stress levels were high among Tunisian doctors with a mean perceived stress score (PSS) of 28.6. One hundred fifteen participants (56.3%) scored for high PSS. This study identified vulnerable subgroups too stress. The female gender, working in the capital and working in critical medicine units were risk factors for high PSS. Managing COVID19 patients was not itself correlated to stress, however social isolation, stigma and luck of access to information were correlated to high stress levels.

**Conclusions:**

Several stressors can affect the well-being of doctors during the COVID-19 pandemic, which can lead to adverse psychosocial outcomes. The findings of this study may guide decision-makers to implement early mental health interventions for doctors.

**Disclosure:**

No significant relationships.

